# An Effective Bearing Fault Diagnosis Technique via Local Robust Principal Component Analysis and Multi-Scale Permutation Entropy

**DOI:** 10.3390/e21100959

**Published:** 2019-09-30

**Authors:** Mao Ge, Yong Lv, Yi Zhang, Cancan Yi, Yubo Ma

**Affiliations:** 1 Key Laboratory of Metallurgical Equipment and Control Technology, Wuhan University of Science and Technology, Ministry of Education, Wuhan 430081, China; ge1656372625@gmail.com (M.G.); yizhang_de@163.com (Y.Z.); meyicancan@wust.edu.cn (C.Y.); Yubo2018.M@gmail.com (Y.M.); 2 Hubei Key Laboratory of Mechanical Transmission and Manufacturing Engineering, Wuhan University of Science and Technology, Wuhan 430081, China

**Keywords:** bearing fault diagnosis, weak fault, multi-component signal, local robust principal component analysis, multi-scale permutation entropy

## Abstract

The acquired bearing fault signal usually reveals nonlinear and non-stationary nature. Moreover, in the actual environment, some other interference components and strong background noise are unavoidable, which lead to the fault feature signal being weak. Considering the above issues, an effective bearing fault diagnosis technique via local robust principal component analysis (LRPCA) and multi-scale permutation entropy (MSPE) was introduced in this paper. Robust principal component analysis (RPCA) has proven to be a powerful de-noising method, which can extract a low-dimensional submanifold structure representing signal feature from the signal trajectory matrix. However, RPCA can only handle single-component signal. Therefore, in order to suppress background noise, an improved RPCA method named LRPCA is proposed to decompose the signal into several single-components. Since MSPE can efficiently evaluate the dynamic complexity and randomness of the signals under different scales, the fault-related single-components can be identified according the MPSE characteristic of the signals. Thereafter, these identified components are combined into a one-dimensional signal to represent the fault feature component for further diagnosis. The numerical simulation experimentation and the analysis of bearing outer race fault data both verified the effectiveness of the proposed technique.

## 1. Introduction

The bearing as an essential element has been widely used in rotating machinery [1,2]. Due to the severe working conditions, such as long and uninterrupted operation, alternating loads, and corrosion, the probability of bearing failure increases greatly, which may cause heavy economic losses or even serious personal injury [3]. Hence, an available diagnosis technique for bearing faults is highly valuable [4]. The bearing faults can be classified into three main types: inner race fault, outer race fault, and rolling element fault [5]. The vibration signals of the bearings are often used for fault diagnosis for their containing abundant equipment operation information [6]. When the bearing faults occur, the corresponding vibration signals will produce periodic impulses, and the feature of this signal behaves in a typical nonlinear and non-stationary nature, which increases in spectral complexity [5,7,8]. In the actual industrial production environment, the signals usually contain some interference vibrations caused by other mechanical components and strong background noise besides useful fault feature. Especially in the early stages of the bearing fault, the fault feature is weak and completely drowned by the strong noise and interferences. Therefore, in order to realize accurate diagnosis of bearing faults, suppressing the background noise and extracting weak fault features from multi-component signals are becoming an urgent work to be solved.

For the diagnosis of the bearing fault signals, some researchers have proposed many methods. Ciabattoni et al. [9] proposed a novel bearing fault classification method by adopting the empirical cumulative distribution functions (ECDFs) of the signal statistical spectral images as the fault feature vectors. The wavelet transform (WT) is the inner product operation between a translated and dialed wavelet basis function and the raw time domain signal. The different feature components and noise in the signal can be separated by the obtained wavelet coefficients [10,11]. Wang et al. [12] extracted the weak fault feature of the rolling element via wavelet packet transform method. Deng et al. [13] presented a novel fault diagnosis method for a motor bearing based on integrating empirical wavelet transform (IEWT) and fuzzy entropy. Xiao et al. [14] applied the wavelet threshold denoising method to effectively de-noise a rolling bearing signal. However, the diagnostic performance of these methods depends on the selection of the wavelet basis functions and the threshold. The Wigner–Ville distribution (WVD) [15] can extract ridges representing feature information from two-dimensional time-frequency plane of the non-stationary signals. Ming et al. [16] applied the cyclic Wiener filter to detect the rolling bearing fault, which uses the spectral coherence theory induced by the second-order cyclostationary signal to extract the weak fault feature. Nevertheless, WVD will produce cross-terms when analyzing multi-component signals. As far as the adaptive signal processing techniques, the empirical mode decomposition method (EMD) and the local mean decomposition (LMD) can be used to deal with nonlinear and nonstationary signals. LMD can decompose any signal into product functions (PFs) representing different feature components [17]. Li et al. [18] introduced a fault diagnosis scheme based on local mean decomposition and an improved multi-scale fuzzy entropy to realize the automatic identification of the bearing fault patterns. LMD is inadequate in processing the signals containing narrow bandwidth components. The multi-component signals can be decomposed into a series of intrinsic mode functions (IMFs) with physical meaning by EMD [19]. Imaouchen et al. [20] employed some demodulation analysis methods based on frequency-weighted energy operator and complementary ensemble empirical mode decompositions to identify the early weak faults of the bearing. Bustos et al. [21] successfully identified the operating state of the gears in high-speed trains through the EMD-based methodology. But the EMD and its improved version always suffer from modal aliasing and boundary effect. Moreover, they are also sensitive to noise. The above research provides rich reference information for bearing fault diagnosis.

It has become quite familiar to view the dynamic characteristics of different features of the raw system by reconstructing the observed time series from nonlinear non-stationary systems into a high dimensional phase space [22,23]. In that way, extracting fault features from high a dimensional phase space is a feasible scheme. The singular value decomposition (SVD) method [24] can decompose the signal trajectory matrix into series interpretable components. The singular values obtained can effectively display the intrinsic properties of different feature components and noise in raw signal. The fault feature can be extracted by setting the singular values representing interference components and background noise to zeros. Currently, the selection of singular values representing fault feature components still depends on experience, which may lead to considerable error. Especially for the early weak faults of the bearings, the singular values representing different feature components are almost impossible to be identified [25]. The classical manifold learning theory holds that the feature component of the signal matrix has a lower intrinsic dimension, which is distributed in a low-dimensional submanifold of a high dimensional phase space [26,27]. As a widely used dimension reduction method, the RPCA can extract this submanifold structure through a rank function constraint based on low-rank matrix approximation (LRMA) and simultaneously suppress background noise through a $l_{0}$-norm regularization strategy [28]. RPCA has proven to be a powerful de-noising tool in image processing, computer vision, and so on [29,30]. However, RPCA is inoperative for the separation of submanifold structures composed of multiple feature components; that is, it cannot process a multi-component signal. Recently, a novel, convex, locally sensitive, low rank matrix approximation (CLSLRMA) method [31] was introduced into the data completion problem, which significantly relaxes the assumption in LRMA that the feature component in matrix has a low-rank submanifold structure. CLSLRMA can decompose a matrix drawn from linear mixture of multiple low-rank manifold subspaces into their respective single subspaces. Hence, it is a feasible way to decompose the trajectory matrix composed of multi-component signals by CLSLRMA.

Permutation entropy (PE) [32] can efficiently evaluate the dynamic complexity and randomness of the signal time series through measuring similarity among the ordinal patterns extracted from the series, which has been widely used for the fault diagnosis of mechanical equipment [33,34]. The dynamic complexity of the bearings will change with the occurrence of faults, resulting in the changing PE values of the vibration signals [33]. However, because of the strong nonlinear and non-stationary characteristic of the acquired mechanical fault feature signals, their complex dynamic characteristics can usually hardly be fully displayed on the original scale, while some important information may also exit over multiple spatial-temporal levels (scales) [32]. Fortunately, based on PE, the multi-scale permutation entropy (MSPE) [32,35] has proven to be one of the most effective methods for which one can explicitly explain the characteristic information from the multiple time scales present in complex time series. Therefore, the MSPE of the signal was adopted in this paper to identify the feature component signal representing the bearing faults.

In this paper, an effective bearing fault diagnosis technique via local robust principal component analysis (LRPCA) and MSPE is introduced. Firstly, on the basis of noise suppression, we proposed an improved RPCA method to decompose the signal into several single-components, which was termed LRPCA. According to CLSLRMA, in the phase space of the weighted matrix associated with different anchor point, we assume that the signal trajectory matrix behaves as a combination of a noise component and a low-dimensional submanifold component, and those submanifold components represent different feature components in the raw signal. LRPCA shows that those submanifold components can be approximated by low-rank matrices through solving a convex program about a weighted combination of the matrix rank constraint function and the $l_{0}$-norm regularization [36,37]. After that, the MPSE was adopted to identify the low-rank matrices corresponding to the fault feature component. Finally, the identified low-rank matrices were transformed into a one-dimensional signal to represent the global approximation of the fault feature component for further diagnosis via weighted Nadaraya–Watson regression model [38]. The processing of the numerical simulation data and the experimental bearing fault data both verified that the proposed technique can provide a great diagnostic performance for bearing faults.

The rest of the paper is organized as follows: Section 2 introduces the theory description, wherein Section 2.1 defines some notations and abbreviations used in this paper; Section 2.2 illustrates the proposed LPRCA method; Section 2.3 describes the MSPE; the detailed step of the proposed bearing fault diagnosis technique is presented in Section 2.4. The analysis of the simulated signal and the experimental signal are performed in Section 3. Section 4 draws the conclusions.

## 2. Theory Description

### 2.1. Notations and Abbreviations

Throughout this paper, we use lowercase letters for scalars, e.g., x; bold and lowercase letters for vectors, e.g., $x\in {\mathbb{R}}^{n_{1}}$; boldface and uppercase letters for matrices, e.g., $X\in {\mathbb{R}}^{n_{1}\times n_{2}}$. The anchor points in X are marked as $e_{i}=(a_{i},b_{i}),\;a_{i}=1,\ldots ,n_{1};\;b_{i}=1,\ldots ,n_{2};\;i=1,\ldots ,m$; the *i*-th element of x can be expressed as $x_{i}$, the (*i*,*j*)-th element of X is denoted as X(i,j) and the *i*-th row of X is expressed as X(i,:). The Hadamard product of two matrix $A\in {\mathbb{R}}^{n_{1}\times n_{2}}$, $B\in {\mathbb{R}}^{n_{1}\times n_{2}}$ is defined as $C=A⊙B\in {\mathbb{R}}^{n_{1}\times n_{2}}$ with its element C(i,j)=A(i,j)B(i,j). The $l_{0}$-norm ${\left\|X\right\|}_{0}$ represents the sum of nonzero elements of X and the $l_{1}$-norm ${\left\|X\right\|}_{1}$ the sum of absolute value of all elements in X. The SVD of X is defined as X=U∑V, where U and V are the left and right singular value matrices; $\Sigma =diag({\left\{\sigma_{i}\right\}}_{1\leq i\leq r})$ represents the singular value matrix. The nuclear norm of X is denoted as ${\left\|X\right\|}_{*}=\sum_{i}\sigma_{i}$.

In the rest of this paper, the following abbreviations are used: RPCA—robust principal component analysis; LRPCA—local robust principal component analysis; MSPE—multi-scale permutation entropy; CLSLRMA—convex local sensitive low rank matrix approximation; SVD—singular value decomposition; SSA—singular spectrum analysis; EMD—empirical mode decomposition; ADMM—alternating direction method of multipliers; and SNR—signal to noise ratio.

### 2.2. Decomposing a Signal into Single-Components via LRPCA

#### 2.2.1. RPCA

The acquired one-dimensional bearing fault signal $x\in {\mathbb{R}}^{n}$ can be converted into a high dimensional signal trajectory matrix $X\in {\mathbb{R}}^{n_{1}\times n_{2}}$ by phase space reconstruction, which is based on a embedding process with the parameter of the embedding dimension $n_{1}$ and the delay time τ (where $(n_{1}-1)\tau +n_{2}=n$) [22]:

(1) $$X=\left[\begin{array}{cccc} x_{1} & x_{2} & \cdots & x_{n_{2}}\\ x_{1+\tau } & x_{2+\tau } & \cdots & x_{n_{2}+\tau }\\ \vdots & \vdots & \cdots & \vdots \\ x_{1+(n_{1}-1)\tau } & x_{2+(n_{1}-1)\tau } & \ddots & x_{n} \end{array}\right]$$

Except for the strong background noise component, X is composed of multiple feature components, which include the fault feature component and the unwanted interfering components. Hence, on the basis of noise suppression, how to separate the useful feature component from these mixed multi-components and back it to the one-dimensional signal to represent the extracted fault feature component was an inevitable task for extracting weak faults in this paper.

**Figure 1:**
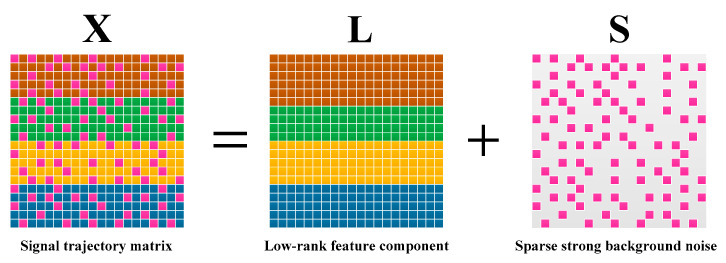
Illustration of the robust principal component analysis (RPCA); A signal trajectory matrix $X\in {\mathbb{R}}^{n_{1}\times n_{2}}$ can be decomposed into a low rank feature component $L\in {\mathbb{R}}^{n_{1}\times n_{2}}$ and a sparse component $S\in {\mathbb{R}}^{n_{1}\times n_{2}}$.

By referring to manifold learning theory, it can be found that the feature component in X has a low-dimensional submanifold structure [26,27]. RPCA can extract this structure by solving the following low-rank matrix and sparse matrix decomposition model (shown as Figure 1):

(2) $$\underset{L,S}{\min\arg}\;rank(L)+\alpha {\left\|S\right\|}_{0},s.t.\;X=L+S$$

where $\alpha =1/\sqrt{\max(n_{1},n_{2})}$ is the optimal weighted parameter. The first term ($L\in {\mathbb{R}}^{n_{1}\times n_{2}}$, r(L)≪r(X)) of this model is a rank constraint function, which uses a low-rank matrix to estimate the low-dimensional submanifold structure, and the second term ($S\in {\mathbb{R}}^{n_{1}\times n_{2}}$) is a regularization strategy, which is mainly used to correct the deviation of matrix data caused by noise. In fault feature extraction, L represents the signal feature component and S captures the noise [28]. Thus, RPCA can effectively separate the feature component of the signal from the noise. However, it can be seen from the theoretical basis of Equation (2) that RPCA can only deal with the matrix data composed of a single feature component and noise, which means this method cannot separate the data containing the multiple feature components.

#### 2.2.2. LRPCA

To tackle the drawback of RPCA in processing the multi-component signals, we proposed a novel LRPCA method based on CLSLRMA to decompose X into several single-components. In LRPCA, the following fundamental assumption is introduced.

Fundamental Assumption: In addition to the background noise signal matrix S, X contains a feature signal matrix L composed of a linear mixture of several feature components. Furthermore, each feature component corresponds to a low-dimensional submanifold structure hidden in the high-dimensional phase space and has a characteristically of low-rank.

**Figure 2:**
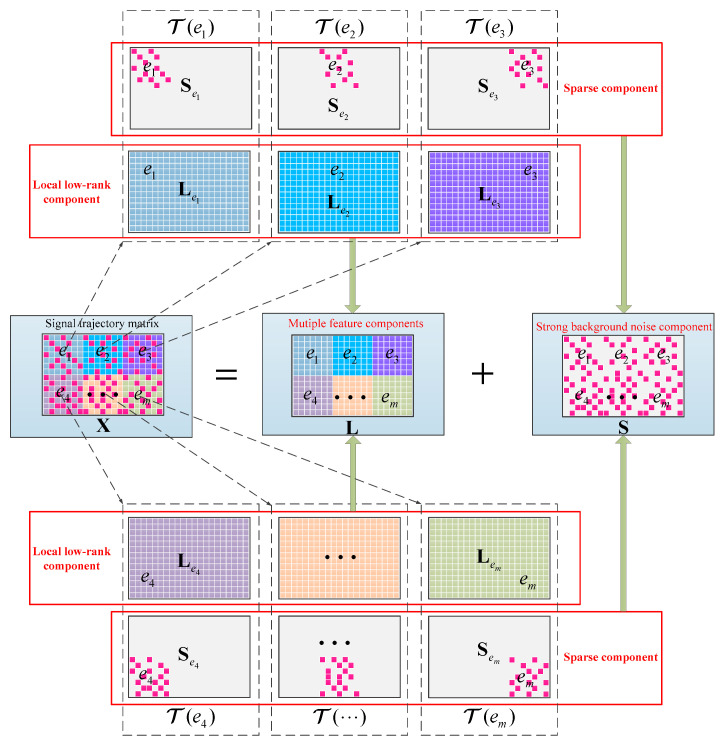
Illustration of the proposed LRPCA method; in the different high-dimensional phase space $T(e_{i})$ associated with the local selected anchor point $e_{i}=(a_{i},b_{i})$, the signal trajectory matrix $X\in {\mathbb{R}}^{n_{1}\times n_{2}}$ can be decomposed into a low rank component $L_{e_{i}}\in {\mathbb{R}}^{n_{1}\times n_{2}}$ and a sparse component $S_{e_{i}}\in {\mathbb{R}}^{n_{1}\times n_{2}}$.

Figure 2 depicts the main ideal of the LRPCA. Specifically, each submanifold structure is generally hidden in the different high-dimensional phase space $T(e_{i})$ associated with the local selected anchor point ($e_{i}=(a_{i},b_{i}),\;a_{i}=1,\ldots ,n_{1};\;b_{i}=1,\ldots ,n_{2};\;i=1,\ldots ,m$), and $T(e_{i})$ is derived from the weighting of X:

(3) $$T(e_{i})=W_{e_{i}}⊙X$$

where $W_{e_{i}}\in {\mathbb{R}}^{n_{1}\times n_{2}}$ is a local weighted coefficient matrix.

Thereafter, $T(e_{i})$ is decomposed into a low-rank component $L_{e_{i}}$ and a sparse component $S_{e_{i}}$. The resulting low-rank matrices $L_{e_{1}},\ldots ,L_{e_{m}}$ represent the low-dimensional submanifolds, which correspond to different feature components in raw signal, that of the fault feature component and the unwanted interfering components. Conclusively, L is expressed as the weighted combination of these local low-rank matrices:

(4) $$L=R_{1}⊙L_{e_{1}}+\cdots +R_{m}⊙L_{e_{m}}$$

where $R_{i}\in {\mathbb{R}}^{n_{1}\times n_{2}}$ is a weighted regression matrix. Thus, the fault-related feature components can be extracted from these low-rank matrices obtained by decomposing L. For this task, a novel local low-rank matrix and sparse matrix decomposition model was proposed to obtain these low-rank matrices from X:

(5) $$L_{e_{i}}=\underset{L_{e_{i}},S_{e_{i}}}{\min\arg}\;rank(L_{e_{i}})+\alpha {\left\|S_{e_{i}}\right\|}_{0},s.t.\;T(e_{i})=L_{e_{i}}+S_{e_{i}}$$

The RPCA is actually a special case of this model when $W_{e_{i}}$ is a unit matrix. Therefore, the de-noising performance of this model can be guaranteed.

• Model Construction and Algorithm Solving

The derivation of the phase space $T(e_{i})$ is the key step in LRPCA. Firstly, a distance function $d((a,b),(a^{\prime },b^{\prime }))$ is defined to describe the similarity between any two elements X(a,b) and $X(a^{\prime },b^{\prime })$ in X. A smaller value of d means the probability of the two elements being in the same phase space is higher. According to the theory of CLSLRMA, The standard incomplete SVD $X=UV^{T}$ [39] is employed to divide d into two independent terms and the arc-cosine function is utilized to calculate these two terms:

(6) $$d(a,a^{\prime })=\arccos(\frac{\langle U(a,:),U(a^{\prime },:)\rangle }{\left\|U(a,:)\right\|\left\|U(a^{\prime },:)\right\|}),\;d(b,b^{\prime })=\arccos(\frac{\langle V(b,:),V(b^{\prime },:)\rangle }{\left\|V(b,:)\right\|\left\|V(b^{\prime },:)\right\|})$$

Then, the following non-parametric smoothing Epanechnikov kernel function [40] is adopted to define the local weighted coefficient matrix $W_{e_{i}}$:

(7) $$W((a,b),(a^{\prime },b^{\prime }))=W(a,a^{\prime })W(b,b^{\prime })=(1-d({\left(a,a^{\prime }\right)}^{2})(1-d{\left(b,b^{\prime }\right)}^{2})$$

The (*i, j*)-element of $W_{e_{i}}$ is expressed as $W(e_{i},(i,j))$. The Epanechnikov kernel function is a typical unilateral quadratic decrement function related to d. Consequently, a lager value of the weighted coefficient means that the weight for X(i,j) belonging to $T(e_{i})$ is bigger. Besides, for different local anchor point $e_{i}$, there theoretically should exist a unique corresponding phase space hidden the single submanifold. However, as the fundamental assumption states, there should be finite number of single submanifold structures in X. Gratifyingly, this function has the mathematical property of changing slowly, which means as long as the similarity of two anchor points $e_{i}$ and $e_{j}$ is large enough, their corresponding phase spaces $T(e_{i})$ and $T(e_{i})$ will have quite high similarity. Moreover, both of those two phase spaces may hide the same single submanifold structure.

Through the above theoretical analysis process, the $T(e_{i})$ satisfying the fundamental assumption was successful created. Finally, $T(e_{i})$ is decomposed to extract the hidden low-rank matrix $L_{e_{i}}$ by solving the Equation (5). Since the discrete combination nature of the $l_{0}$-norm and rank function, the solution of this model is an non-deterministic polynomial (NP)-hard problem [41]. Take this into consideration, some recent studies [36,37,42,43,44,45] pointed out that an equivalent convex optimization program of this model can be obtained from the convex hull of the two constraints; that is, the $l_{1}$-norm and the nuclear norm are employed to replace those two constraints respectively:

(8) $$L_{e_{i}}=\underset{L_{e_{i}},S_{e_{i}}}{\min\arg}{\left\|L_{s_{i}}\right\|}_{*}+\alpha {\left\|S_{e_{i}}\right\|}_{1},\;s.t.\;T(s_{i})=L_{e_{i}}+S_{e_{i}}$$

The solution of Equation (8) is a typical convex optimization problem, whose minimizer is globally unique [43]. Algorithm 1 provides a precise and convergent solution to this equation via ADMM [44,46]. Note that steps 3 and 4 are both convex problems, they both have closed-form solutions via singular value thresholding operator [47].

**Algorithm 1** solve (8) by ADMM  **Input:** signal trajectory matrix $X\in {\mathbb{R}}^{n_{1}\times n_{2}}$;   **Parameter:** number of anchor points: q; regularization parameter: $\alpha =1/\sqrt{\max(n_{1},n_{2})}$;   **for all** i = 1:m, **parallel do**;    **1.** select $e_{i}(a_{i},b_{i})$ uniformly in X, and calculate $W_{e_{i}}$ by Equation (7);    **2.**
$T(e_{i})=W_{e_{i}}⊙X$;    **Initialize:**
$L_{e_{i}}^{0}=S_{e_{i}}^{0}=Y^{0}=0$**,**
$\gamma_{0}=e^{-3}$**,**
$\gamma_{\max}=e^{10}$**,**
μ=1.1**,**
ε=1e−8;    **while** not converged do;     **3.** fix the others and update $L_{e_{i}}^{k+1}$ by:$$L_{e_{i}}^{k+1}=\underset{L_{e_{i}}}{\arg\min}:{\left\|L_{e_{i}}\right\|}_{*}+\frac{\gamma_{k}}{2}{\left\|L_{e_{i}}+S_{e_{i}}^{k}-T(e_{i})+Y^{k}\right\|}_{F}^{2}$$     **4.** fix the others and update $S_{e_{i}}^{k+1}$ by:$$S_{e_{i}}^{k+1}=\underset{S_{e_{i}}}{\arg\min}:\lambda {\left\|S_{e_{i}}\right\|}_{1}+\frac{\gamma_{k}}{2}{\left\|L_{e_{i}}^{k+1}+S_{e_{i}}-T(e_{i})+Y^{k}\right\|}_{F}^{2}$$     **5.** update Lagrange multiplier Y: $Y^{k+1}=Y^{k}+T(e_{i})-L_{e_{i}}^{k+1}-S_{e_{i}}^{k+1}$;     **6.** update τ**:**
$\tau_{k+1}=\min(\mu \tau_{k},\tau_{\max})$;     **7.** check the convergence conditions:$${\left\|L_{e_{i}}^{k+1}-L_{e_{i}}^{k}\right\|}_{\infty }\leq \varepsilon ,{\left\|S_{e_{i}}^{k+1}-S_{e_{i}}^{k}\right\|}_{\infty }\leq \varepsilon ,{\left\|L_{e_{i}}^{k+1}+S_{e_{i}}^{k+1}-T(e_{i})\right\|}_{\infty }\leq \varepsilon$$   **end;**  **end;**  **output:**
$L_{e_{1}},\cdots ,L_{e_{m}}$.

Through Algorithm 1, we can obtain m low-rank matrices corresponding to different feature components in the raw signal. Besides, it needs to be emphasized that the performance of the final fault diagnosis is highly susceptible to the location selection and the number of the anchor points. Once the chosen anchor points are inappropriate, there may be multiple low-rank matrices corresponding to the same feature component. More seriously, the fault-related low-rank matrices may be completely missed. In view of the above problems, for the selection of the anchor points, we do not have a good solution for now. But, in the process of analyzing the experimental data, the following two principles are feasible. The first one is that m should be large enough to ensure that the low-rank matrices corresponding to all feature components can be extracted specifically. This can be explained from the perspective of the probability. When the number of the anchor points is more, the probability of the extracted fault-related low-rank matrices is higher. However, this inevitably requires a lot of computing time. The other one is that these anchor points should be uniformly chosen from the elements set and the distance between any two anchor points should be made large enough. This principle is to make extracted low-rank matrices corresponding to the different feature components differ as much as possible. In the numerical simulation experiment, since the raw simulated signal contained three feature components, and we found that when m was set as six, the final decomposition performance was quite good. Additionally, when analyzing the experimental signal, it was found that m=6 is also appropriate. Therefore, according to the analysis results of the experimental data, we assumed that the number of the main feature components in the acquired bearing fault signal generally would not exceed three, and set m to be twice that number; that is m=6.

• Global Approximation of Fault Feature Component

These low-rank matrices can be backed to m one-dimensional component signals by inverse transform. In Section 2.2, it will show that the one-dimensional components related to fault feature can be identified from these signal through the MPSE characteristic of the signal. Thus, there are o (o<m) identified one-dimensional signals and their corresponding low-rank matrices $L_{e_{1}},\ldots ,L_{e_{o}}$. These low-rank matrices are actually local sensitive, which can only be used to describe the fault feature information contained in the corresponding low-dimensional submanifolds. Hence, the Nadaraya-Watson regression model [38] is adopted to combine these low-rank matrices into a global approximation ${\hat{L}}_{f}\in {\mathbb{R}}^{n_{1}\times n_{2}}$ of the fault feature component, which can be expressed as:

(9) $${\hat{L}}_{f}=\sum_{i=1}^{o}\frac{W_{e_{i}}}{\sum_{j=1}^{m}W_{e_{j}}}⊙L_{e_{i}}$$

Note that if o=m, this equation is equivalent to Equation (4). Thus, the estimator of L can be obtained, which is actually a de-noising process. Thereby, we returned ${\hat{L}}_{f}$ to the one-dimensional time series ${\hat{l}}_{f}\in {\mathbb{R}}^{n}$, which is the expected fault feature signal. Hence, the task to suppress the strong background noise and extract the fault feature signal was completed.

### 2.3. Identification of Bearing Fault-Related Signal through MSPE

#### 2.3.1. Basic Theory of MSPE

**Figure 3:**
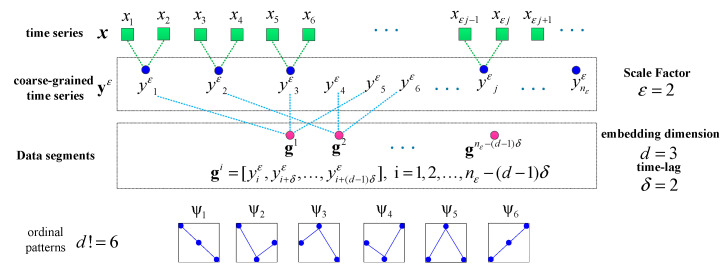
Illustration of the coarse-grained and data segments of the time series with ε=2, d=3, and δ=2, as well as the all d!=6 type of ordinal patterns.

The MPSE can efficiently evaluate the dynamic complexity and randomness of the time series under different scales. The calculation of the MSPE depends on three parameters: scale factor ε, embedding dimension d and time-lag δ [32,36]. For a signal time series $x=[x_{1},x_{2},\cdots ,x_{n}]$, the main calculation steps can be divided as follows:

(1) Transform x into a successive coarse-grained time series $y^{\varepsilon }\in {\mathbb{R}}^{n_{\varepsilon }}$ ($n_{\varepsilon }=[n/\varepsilon ]$) by averaging the time data points in x with the given non-overlapping time slice of the increasing length, ε. Then, each element of $y^{\varepsilon }$ is defined as:
  (10) $$y_{i}^{\varepsilon }=\frac{1}{\varepsilon }\sum_{j=(i-1)\varepsilon +1}^{i\varepsilon }x_{j},i=1,2,\cdots ,n_{\varepsilon }$$
(2) For each coarse-grained time series $y^{\varepsilon }$, the PE value needs to be calculated. Firstly, $y^{\varepsilon }$ is cut into a series of data segments through d and δ:
  (11) $$g^{i}=[y_{i}^{\varepsilon },y_{i+\delta }^{\varepsilon },\ldots ,y_{i+(d-1)\delta }^{\varepsilon }],i=1,2,\ldots ,n_{\varepsilon }-(d-1)\delta$$
  There will be $n_{\varepsilon }-(d-1)\delta$ data segments in total. Then, there are d! different types of ordinal patterns ($\psi_{i},i=1,\ldots ,d!$) in the data segments. Then, count the frequency of each pattern and denote them as $f{(\psi }_{i}),i=1,\ldots ,d!$. Thus, the relative frequency of each pattern can be written as:
  (12) $$p(\psi_{i})=f(\psi_{i})/(n_{\varepsilon }-(d-1)\delta )$$
  Finally, the PE of $y^{\varepsilon }$ is expressed as:
  (13) $$P(\varepsilon )=-\sum_{i=1}^{d!}p(\psi_{i})\log_{2}p(\psi_{i})$$
  For convenience, we normalize P(ε) by dividing its maximum value $\log_{2}d!$:
  (14) $$0\leq P(\varepsilon {)/\log}_{2}d!\leq 1$$
(3) The PE values of different coarse-grained time series can be obtained and plotted as a function of the scale factors. The vector P=[P(1),P(2),…], formed by the set of PE values, is the MSPE of the original time series. Figure 3 shows the process of coarse granulation and the data segmentation of a time series.

#### 2.3.2. Mathematical Model of Bearing Fault Feature Signal

The bearing fault feature signal can be modeled as the combination of finite pulsed excitations [8,48,49,50]:

(15) $$x(t)=a(t)\sum_{i=0}^{I}b_{i}(t)\cos(2\pi f_{e}t-c_{i}(t)+\theta_{i})$$

where $b_{i}(t)$, c(t), and $a_{i}(t)$ indicate the amplitude modulation component, the frequency modulation component, and the modulation effect caused by vibration transfer path, respectively. $f_{e}$ and $\theta_{i}$ indicate the system resonance frequency and the initial phase, respectively.

$b_{i}(t)$ and $c_{i}(t)$ can be expressed as:

(16) $$b_{i}(t)=B_{i}e^{-\xi (t-iT_{d}-\upsilon_{i})}u(t-iT_{d}-\upsilon_{i})$$

(17) $$c_{i}(t)=\sum_{l=1}^{L}C_{il}\sin(2\pi lf_{c}t+\theta_{il})$$

where ξ, $f_{c}$, and $T_{d}$ indicate the resonance attenuation coefficient, the fault feature frequency, and the time period of fault, respectively. u(t) represents a unit step function. $\upsilon_{i}$ and $\theta_{il}$ represent the random slip of the *i-*th pulse and the initial phase, respectively. $B_{i}$ and $C_{il}$ are amplitudes.

Generally, the vibration sensor is mounted at the bearing seat, which is fixed with the outer race. In the case of the rolling element fault or the inner race fault, the vibration propagation generated by the signal transfer path from the fault location to the sensor is varies with time, resulting in an amplitude modulation effect in the signal:

(18) $$a(t)=A[1+\cos(2\pi f_{r}t)]$$

where $f_{r}$ indicates the rotation frequency of the shaft where the fault bearing is located, and C is a constant.

In the case of the outer race fault, the vibration propagation only has a scaling effect on the signal amplitude due to the transfer path being fixed:

(19) a(t)=A

**Figure 4:**
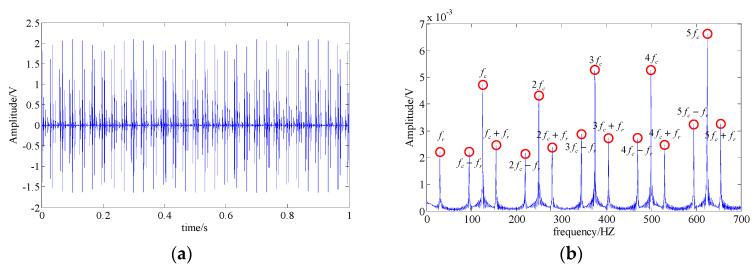
A simulated fault feature signal of a bearing’s inner race; (**a**) signal waveform; (**b**) signal spectrum.

**Figure 5:**
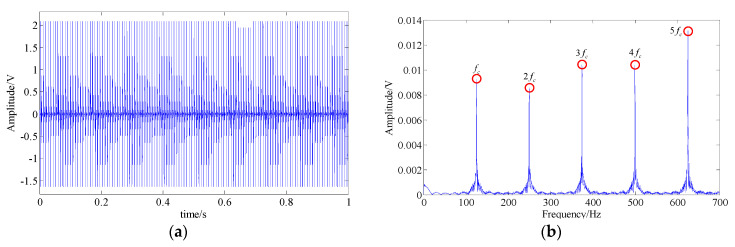
The simulated fault feature signal of bearing outer race; (**a**) signal waveform; (**b**) signal spectrum.

One accepted approach to fault identification is to identify the fault-related frequency contents from the signal spectrum. For example, Figure 4 shows the waveform and the spectrum of a simulated bearing’s inner race fault signal, and the related frequency content includes: the fault feature frequency $f_{c}$, its harmonic frequencies $nf_{c}$, the rotational frequency $f_{r}$, and the modulated side band formed by their combination $nf_{c}\pm f_{r}$; Figure 5 shows the waveform and the spectrum of a bearing outer race fault signal. The frequency content includes: the fault feature frequency $f_{c}$ and its harmonic frequencies $nf_{c}$. It can be observed that nonlinear and non-stationary nature of these fault signals increases the complexity of the spectrum. Especially in the fault of inner race, where the complex modulation side band appears. As a result, the dynamic characteristic of the inner race fault should be more complex than that of outer race fault.

#### 2.3.3. Identification of Fault-Related Components through MSPE

When faults occur in a mechanical system, such as gear or bearing parts, the MSPE value of the vibration signal will change, and the value varies with different type of the faults. Thus, MSPE can be adopted to identify the fault-related components.

**Figure 6:**
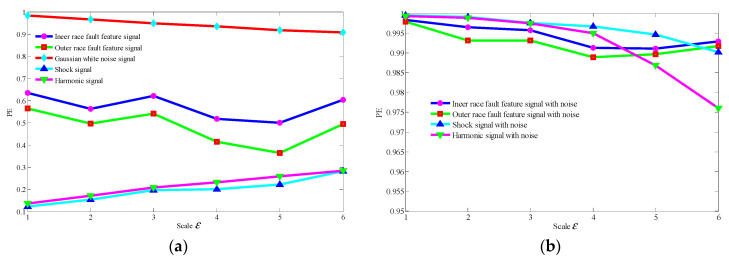
The MSPE of different types of simulated signals; (**a**) five types of signal: the two bearing fault feature signals shown as Figure 4 and Figure 5, a harmonic signal with the main frequency of 150 Hz, a shock signal with the main frequency of 150 Hz, and a Gaussian white noise signal; (**b**) the MSPEs of four feature signals in Figure 6a mixed with Gaussian white noise (signal to noise ratio (SNR) = −5).

The common interference components in bearing signals include the shock signals generated by other parts and the harmonic signals. Therefore, without loss of generality, we discuss the MSPE characteristics of different types of components bearing fault signals, including the fault feature signals of the bearing’s inner race (shown as Figure 4) and the bearing outer race (shown as Figure 5), a harmonic signal, a shock signal, and a Gaussian white noise signal. The results of these five type signals are shown as Figure 6a. The results demonstrate that the MSPE value of a regular time series, such as harmonic signal or shock signal, is smaller, the examples being basically below 0.3; in contrast, since advent of the complex dynamic characteristic, the MPSE values of the bearing fault feature signals are larger, ranging from 0.4 to 0.7. In addition, it can be observed that the MSPE value of the inner race fault is larger than that of the outer race. In particular, the randomness of the noise signal is the strongest with the MSPE value more than 0.9, which proves that the MSPE is sensitive to noise.

According to the above analysis results, we can set a threshold range (0.5–0.85) of the MSPE value or select the larger value (but no more than 0.9) to identify the components related to bearing fault feature from the q one-dimensional component signals obtained by LRPCA.

It needs to be emphasized that although MSPE has a good anti-noise ratio, it may not be able to effectively identify the early weak fault feature signals under strong background noise. Figure 6b shows the MPSE of the four feature signals in Figure 6a, after Gaussian white noise with a SNR of −5 is added. Due to the existence of the strong background noise, the randomness of signals is greatly enhanced, resulting in the MSPE values of the four feature signals increasing to more than 0.9 and mixing together. As a result, the fault feature components are impossible to be identified. Therefore, it is an urgent problem to reduce the noise before identifying the weak fault feature components.

### 2.4. The Process of the Effective Fault Diagnosis Technique

**Figure 7:**
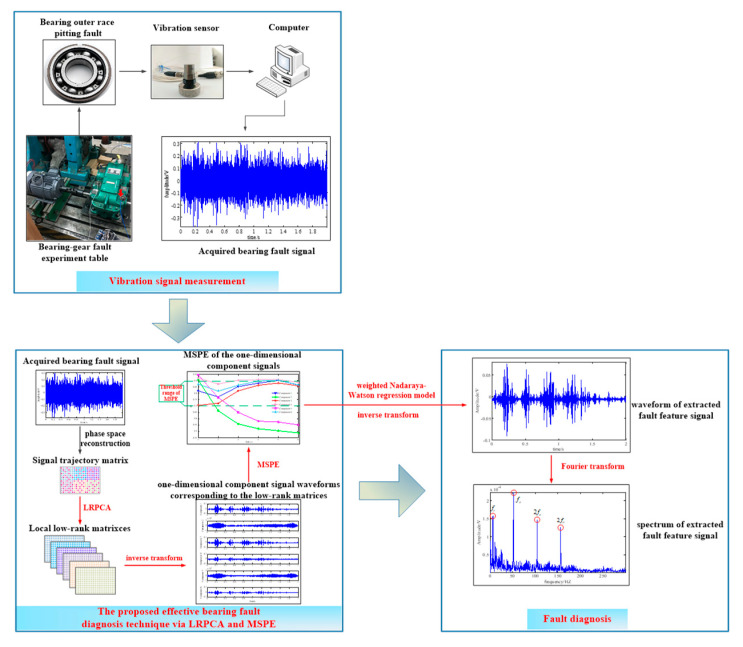
The flowchart of the proposed effective fault diagnosis technique via LRPCA and MSPE.

Figure 7 depicts the flowchart of the proposed effective fault diagnosis technique based on the above theoretical description. And the main steps are summarized as follows:

(1) Using the proposed LRPCA method to decompose the trajectory matrix consisting of the acquired bearing fault signal into multiple low-rank matrices and to suppress the noise synchronously;
(2) Convert the low-rank matrices obtained into one-dimensional component signals by inverse transformations and identify the fault-related components from these signals through the MPSE characteristic of the signal;
(3) Using the weighted Nadaraya–Watson regression model and inverse transform to combine the low-rank matrices corresponding to identified components into a one-dimensional signal to represent the extracted fault feature component;
(4) Confirm the bearing fault by identifying the fault-related frequency contents from the signal spectrum.

## 3. Experiments

### 3.1. Numerical Simulation Experiment

**Figure 8:**
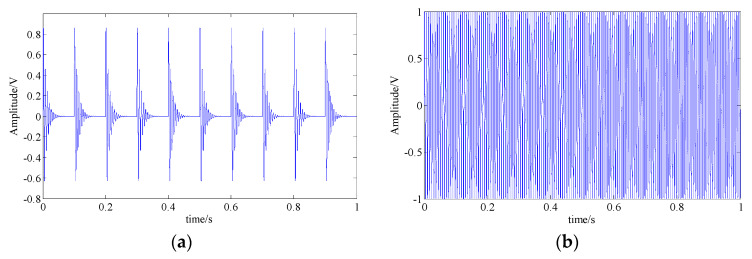
The simulated shock signal and harmonic signal; (**a**) shock signal waveform; (**b**) harmonic signal waveform.

In order to verify the diagnostic performance of the proposed technique, without loss of generality, a simulated signal composed of multiple components was generated:

(20) $$x=x_{1}(t)+x_{2}(t)+x_{3}(t)+n(t)$$

where $x_{1}(t)$ is the simulated fault feature signal of a bearing’s inner race, as shown in Figure 4, and its detailed parameters are listed in Table 1. $x_{2}(t)$ and $x_{3}(t)$ are the interferences of a shock signal and a harmonic signal, respectively. Figure 8a,b shows the waveforms of these two signals and both of their feature frequencies are 150 Hz. n(t) represents the strong background white Gaussian noise. The signal sampling frequency and the sampling point are $f_{s}=$ 20,000 and n= 20,000, respectively.

**Figure 9:**
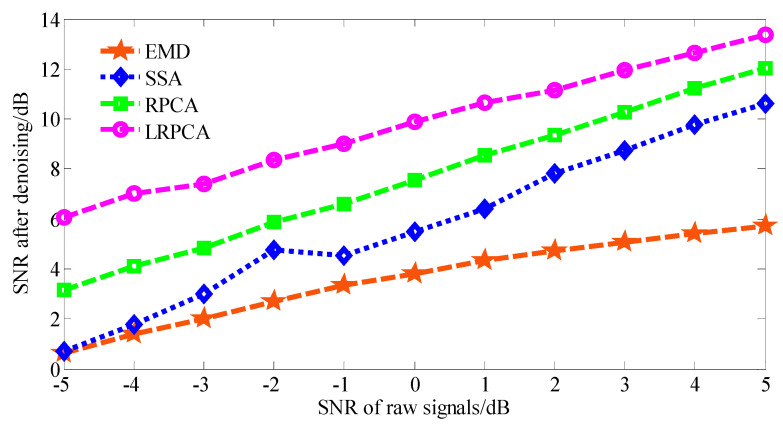
Comparison of the de-nosing performance of four methods when white Gaussian noise of varying SNR is added to the multi-component signal.

Firstly, the de-noising performance of the proposed LRPCA method was tested. The strong background noise with SNR from −5 to 5 db was added to the simulated signals to imitate the early weak fault. For visually displaying the de-noising performance of LRPCA, the methods of RPCA, SSA, and EMD were employed to make a comparative analysis. The phase space reconstruction parameters used in LRPCA, RPCA, and SSA were all set as $n_{1}=$ 200 and τ= 100. EMD employed the energy difference tracking method [51] to select desired IMF components. The hard threshold method [52] was adopted in SSA to select the best combination of singular values to reconstruct the signal. Figure 9 illustrates the de-noising result of the above four methods. In the vertical axis of the graphics, the higher SNR value indicates a better de-noising performance. It is clear that the proposed LRPCA method can provide the best noise suppression performance.

**Figure 10:**
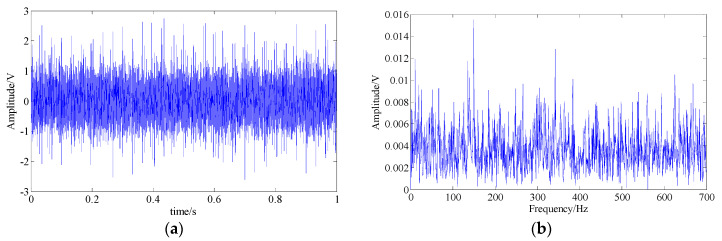
The simulated multi-component signal contains a strong white Gaussian noise with the SNR of −5 db; (**a**) signal waveform; (**b**) signal spectrum.

Then, the fault feature extraction performance of the proposed technique was investigated. In this experiment, a noise with a SNR of −5 db was added into the simulation signal. Figure 10 shows the waveform and the spectrum of the noise-signal mixture. It can be seen that the fault feature is completely submerged by noise and the other interferences, which inevitably increases the difficulty of recognizing the fault feature. The methods of wavelet shrinkage denoising [14], basis pursuit denoising [53], EMD, and SSA were selected for comparative analysis.

**Figure 11:**
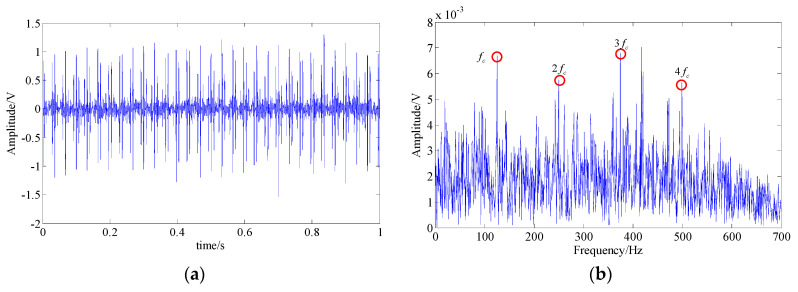
The analysis result of the wavelet shrinkage denoising method; (**a**) waveform of the extracted fault feature signal; (**b**) spectrum of the extracted fault feature signal.

In the wavelet shrinkage denoising method, the decomposition layer was selected as 4 and the wavelet basis function was set as “db15.” The waveform and the spectrum of the final extracted fault feature signal are shown in Figure 11. In the resulting spectrum, the fault feature frequency ($f_{c}$) and some of its harmonic frequencies ($2f_{c}$−$4f_{c}$) can be found. However, due to a large number of interference components and strong background noise, the identification of these fault-related frequencies was seriously affected, and the modulation sidebands were completely submerged. hence, this method was insufficient in feature extraction of a simulated signal.

**Figure 12:**
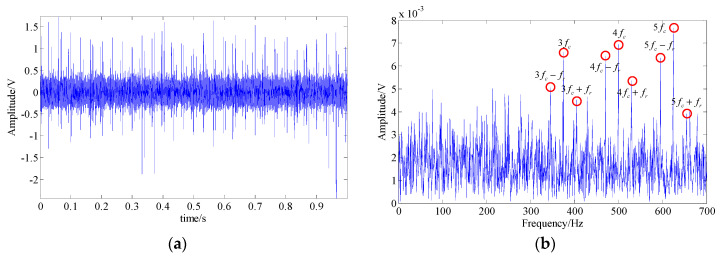
The analysis result of the basis pursuit denoising method; (**a**) waveform of the extracted fault feature signal; (**b**) spectrum of the extracted fault feature signal.

In the basis pursuit denoising method, we adopted the compressed sensing reconstruction algorithm [54] to extract the fault feature signal and Figure 12 shows the final analysis result. In the resulting spectrum, the Fault harmonic frequencies ($3f_{c}$−$5f_{c}$) and their modulated sidebands ($3f_{c}\pm f_{r}$, $4f_{c}\pm f_{r}$, and $5f_{c}\pm f_{r}$) could be identified. But, there were still a lot of interference frequency components and strong background noise, leading to the fault of the signal not being directly determined.

**Figure 13:**
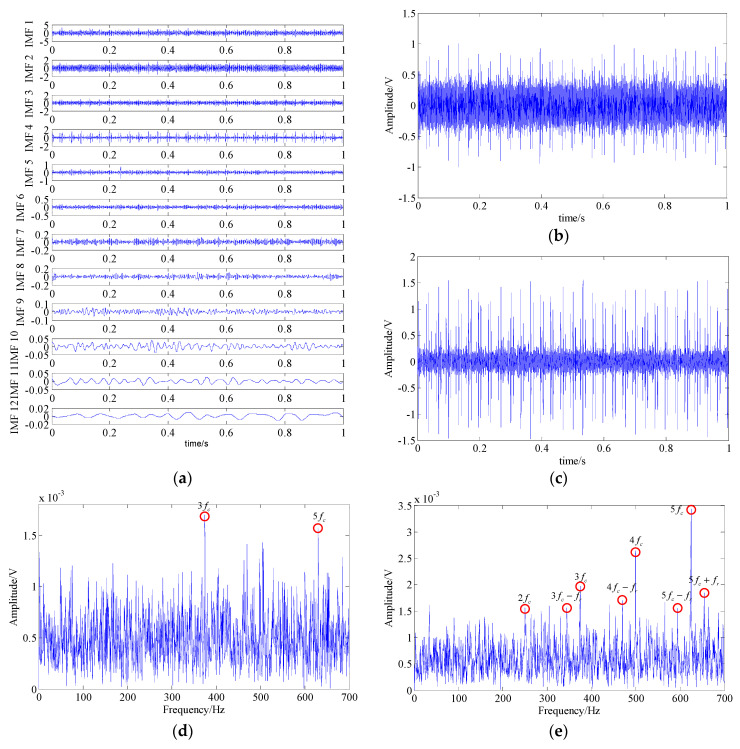
The analysis result of the EMD method; (**a**) waveforms of top 12 IMFs; (**b**,**d**) waveform and spectrum of IMF 3; (**c**,**e**) waveform and spectrum of IMF 4.

In the EMD, fourteen IMFs can be obtained by decomposing the signal and the waveforms of their top twelve are shown in Figure 13a. By applying Fourier transform to these IMFs, in the resulting spectrums of IMF3 and IMF4, partial fault-related frequency contents can be found; i.e., fault harmonic frequencies ($2f_{c}$−$5f_{c}$) and the modulated sidebands ($3f_{c}-f_{r}$, $4f_{c}-f_{r}$, and $5f_{c}+f_{r}$). The waveform and the spectrum of these two IMFs are shown in Figure 13b–e. However, there were still plenty of interference frequency components and noise, which are adverse to the identification of fault feature.

**Figure 14:**
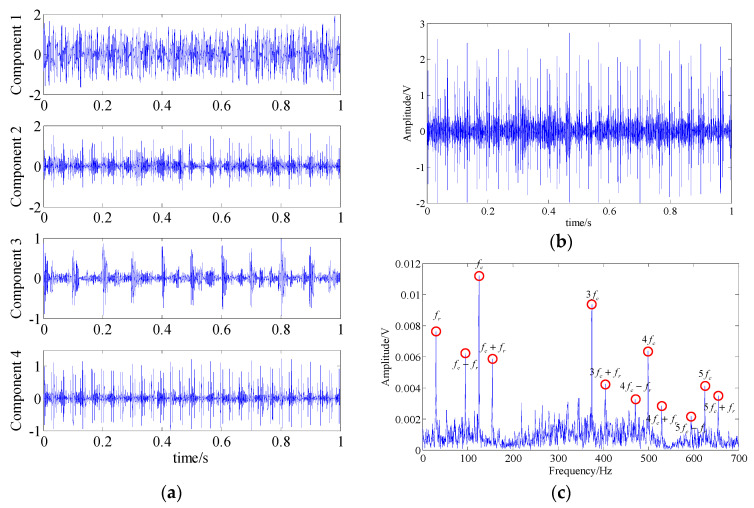
The analysis result of the SSA method; (**a**) waveform of the one-dimensional component signals; (**b**) waveform of the extracted fault feature signal; (**c**) spectrum of the extracted fault feature signal.

In the SSA, by setting the threshold value of the energy of singular value to reach 95% of the total, four singular subspaces representing different feature components can be obtained. Figure 14a illustrates the waveforms of the corresponding one-dimensional feature component signals of these four subspaces obtained through inverse transform. Then, through similarity analysis, it was concluded that components 2 and 4 had higher similarity with the raw fault feature signal. Thus, the signal obtained by adding them represents the extracted fault feature component. The waveform and the spectrum of this extracted signal are shown in Figure 14b,c. In the spectrum, some fault-related frequency contents could be found. But, similar to the analysis result of EMD, there were also some interference frequency components and noises, which may have had an adverse effect on the final diagnosed result.

**Figure 15:**
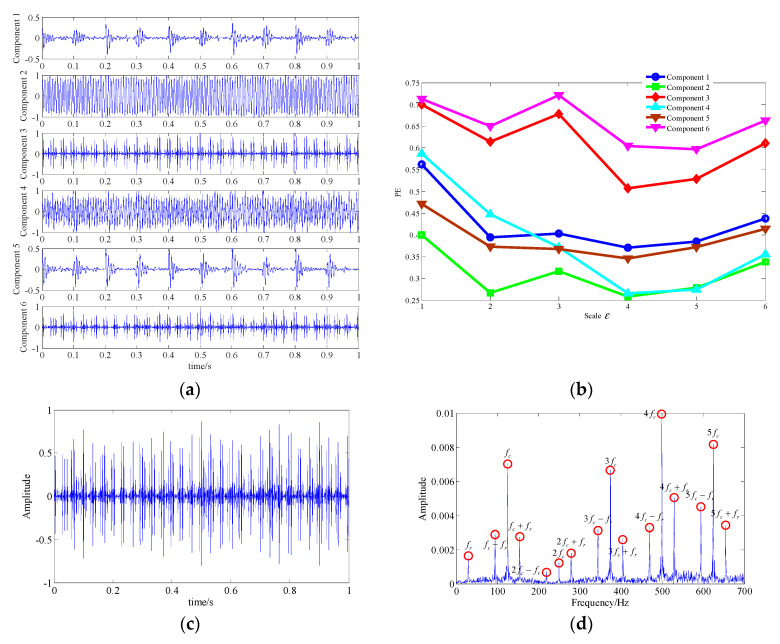
Analysis result of the proposed technique; (**a**) waveform of the one-dimensional component signals; (**b**) the MSPE of the components; (**c**) waveform of the extracted fault feature signal; (**d**) spectrum of the extracted fault feature signal.

The diagnosed result of the proposed technique is shown in Figure 15. Figure 15a shows the waveforms of the one-dimensional component signals obtained by LRPCA, which correspond to the six low-rank matrices. Additionally, the MSPEs of those components are displayed in Figure 13b. It can be observed that the MSPE values of component 3 and 6 are relatively higher and evenly distributed between 0.5 and 0.8. Thus, we could combine the low-rank matrices corresponding to those two components into a one-dimensional signal to represent the extracted fault feature signal. Figure 15c,d show the waveform and the spectrum of this extracted signal. All fault related frequencies were clearly discernable in the spectrum. Furthermore, it can be observed that the interference frequency components and the background noise were basically eliminated. According to the above analysis information, we can undoubtedly confirm that the fault occurred at the inner race.

The above simulation results indicate that EMD and SSA perform when decomposing a complicated multi-component signal into finite single-components. However, their anti-noise ability against strong noise is insufficient and there are still some interference components in the final extracted feature signals. On the contrary, the proposed technique is more effective in eliminating the strong background noise and extracting the weak fault feature component, and its diagnostic performance for the simulated signal is obviously better than the other two methods.

### 3.2. Experimental Signal Analysis

**Figure 16:**
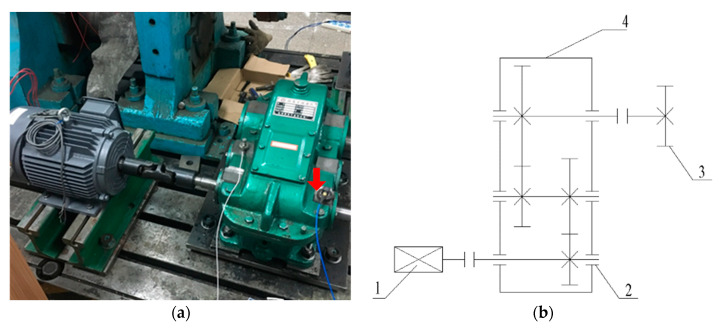
Bearing-gear fault experiment table; (**a**) physical photograph; (**b**) structural drawing: 1—AC motor; 2—the mounting position of fault bearing; 3—magnetic powder brake; 4—gearbox.

The vibration signal acquired on the spot is more complex than the simulation signal. In order to further verify the practicability of the proposed technique, a pitting fault signal of the bearing’s outer race sampling from a bearing-gear fault’s experimental table was analyzed. Figure 16 shows the experimental table, which consists of an AC motor, couplings, a gearbox with two pairs of meshing gears, and a magnetic powder brake. The test bearing is a single-row tapered roller bearing of the type of 32206 and its fault was handled by electrical discharge machining (EDM) method. The red arrow in Figure 16a displays the mounting position of the bearing. The experiment parameter was listed in Table 2. The vibration data of this experiment are measured by PCB acceleration sensor in the vertical direction of the bearing.

**Figure 17:**
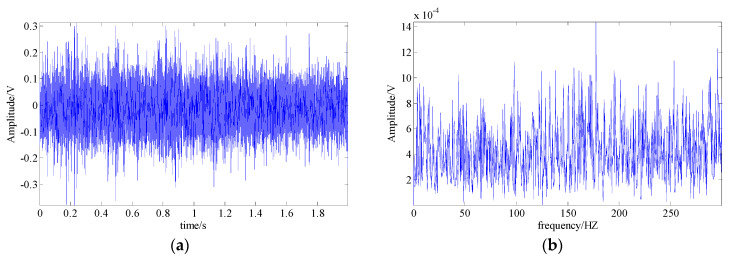
The acquired bearing fault signal; (**a**) signal waveform; (**b**) signal spectrum.

Figure 17 is the diagram of the waveform and the spectrum of the acquired signal. It can be seen that the weak fault feature was impossible to be identified. Then, the proposed technique was utilized to diagnose the signal, and the methods of wavelet shrinkage denoising, basis pursuit denoising, EMD, and SSA were chosen for a comparative analysis.

**Figure 18:**
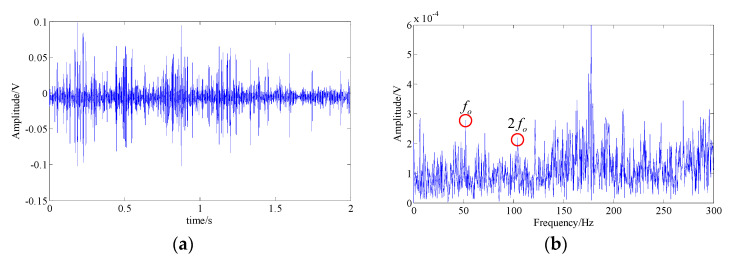
Analysis result of the wavelet shrinkage denoising method; (**a**) waveform of the extracted fault feature signal; (**b**) spectrum of the extracted fault feature signal.

**Figure 19:**
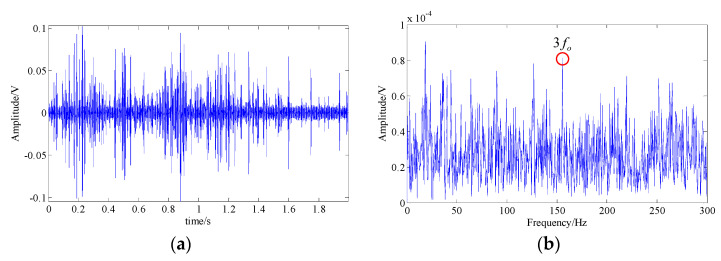
Analysis result of the basis pursuit denoising method; (**a**) waveform of the extracted fault feature signal; (**b**) spectrum of the extracted fault feature signal.

The analysis results of the wavelet shrinkage denoising method and basis pursuit denoising method are shown as Figure 18 and Figure 19, respectively. In their result spectrums, although the fault characteristic frequency ($f_{o}$) and its harmonic frequencies ($2f_{o}$−$3f_{o}$) can be found, the identification of these fault-related frequencies is seriously affected by a large number of interference components and strong background noise. Therefore, the fault type of bearing cannot be directly determined from these analysis results.

**Figure 20:**
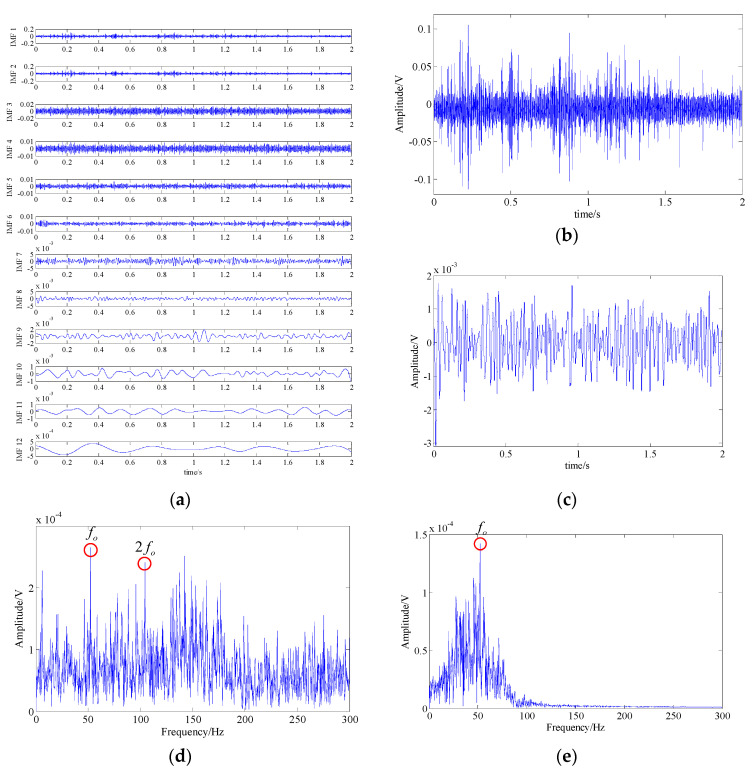
Analysis result of the EMD method; (**a**) waveforms of top 12 IMFs; (**b**,**d**) waveform and spectrum of IMF 1; (**c**,**e**) waveform and spectrum of IMF 8.

Figure 20a displays the waveforms of top 12 IMFs obtained through EMD. By applying the Fourier transform to them, the fault-related frequency contents ($f_{o}$ and $2f_{o}$) can be found in the spectrums of IMF 1 and IMF 8, which are shown in Figure 20b,d,e. However, that fault feature information is hard to be recognized due to the presence of many interference frequency components and strong background noise. Therefore, it is difficult to determine whether the outer race of the bearing is faulty.

**Figure 21:**
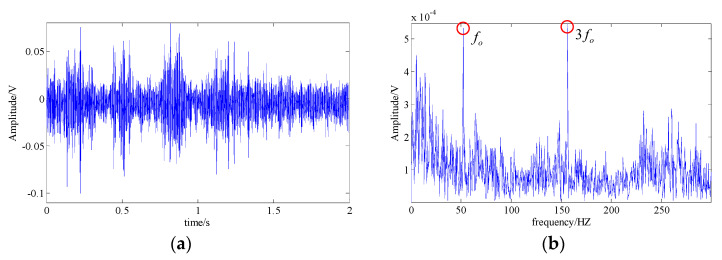
Analysis result of the SSA method; (**a**) waveform of the extracted fault feature signal; (**b**) spectrum of the extracted fault feature signal. The fault feature extraction results of SSA. The peaks of the fault feature frequency ($f_{o}$) and its triple frequency ($3f_{o}$) were obvious in the spectrum. But there are still many interference peaks and noise, which affect the identification of the fault feature. These above analysis results indicate that neither EMD nor SSA can provide a good fault diagnosis performance for the experimental fault signal.

Figure 21 shows the fault feature extraction result of SSA. The peaks of fault feature frequency ($f_{o}$) and its triple frequency ($3f_{o}$) were obvious in the spectrum. But there are still many interference peaks and noise, which affects the identification of fault feature. These above analysis results indicate that neither EMD nor SSA can provide good fault diagnosis performances for the experimental fault signal.

**Figure 22:**
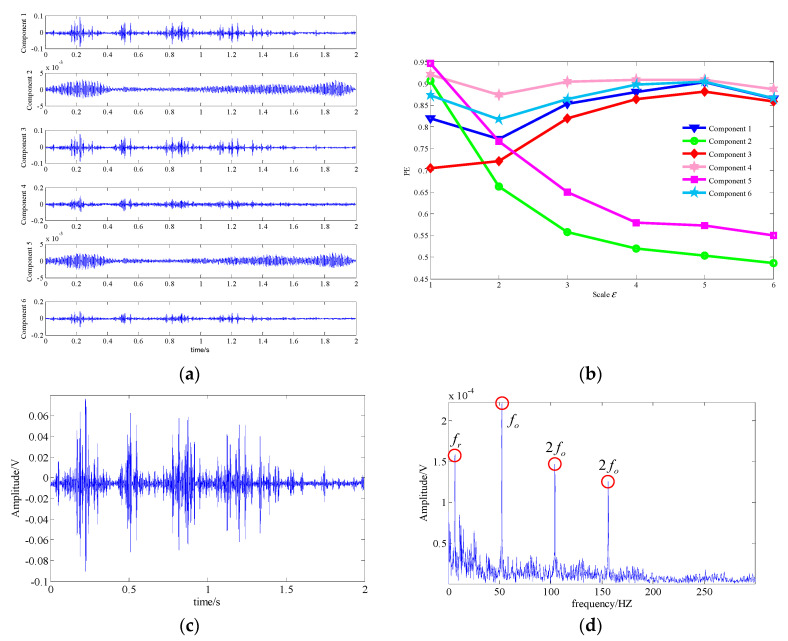
Analysis result of the proposed technique; (**a**) waveform of the one-dimensional component signals; (**b**) the MSPE of the components; (**c**) waveform of the extracted fault feature signal; (**d**) spectrum of the extracted fault feature signal.

The proposed technique was utilized to diagnose the signal. Figure 22a shows the waveforms of the one-dimensional component signal corresponding to the six low-rank matrices obtained by LRPCA. Furthermore, their MSPE values were displayed in Figure 22b. It can be observed that the MSPE values of component 1, 3, 4, and 6 are relatively higher. Meanwhile, the MSPE value of component 4 was basically above 0.9, which may be the feature signal of other component with more complex dynamics characteristic. The MSPE values of components 1, 3, and 6 ranged from 0.7 to 0.9, and their trends were similar, so we combined the low-rank matrices corresponding to these three components into a one-dimensional feature signal representing the extracted the fault feature signal. Figure 22c,d show the waveform and the spectrum of this extracted signal. In the spectrum, the rotation frequency ($f_{r}$), the fault feature frequency ($f_{o}$), and its double frequency ($2f_{o}$) and triple frequency ($3f_{o}$) can be easily found. Moreover, it can be seen that the energy of the noise was suppressed at a low level and there were a few interference frequency components, which had little effect on the final diagnosed result. Consequently, we could confirm that the outer race of bearing had fault. These analysis results demonstrate that the proposed technique can effectively suppress the strong background noise and extract weak fault feature component from the multi-component signal, which means the proposed technique can provide a great diagnostic performance when dealing with the experimental signal.

## 4. Conclusions

In general, the bearing’s weak fault feature exhibits the nature of nonlinear and non-stationary, which is hard to be extracted under the situation of existing strong background noise and interference components. In considering this problem, an effective bearing fault diagnosis technique via LRPCA and MSPE was introduced in this paper. The LRPCA can decompose the signal trajectory matrix into multiple low-rank matrices, and meanwhile, suppress the noise. The MSPE was used to identify the low-rank matrices corresponding to bearing’s fault feature. Thereafter, those identified low-rank matrices were combined into a one-dimensional signal to represent the extracted fault feature component for further fault diagnosis. The principle and the effectiveness of this technique was verified by the analysis of both the simulation signal and the acquired bearing fault signal. The analysis results indicate that the proposed technique can effectively detect and locate the bearing faults accurately.

The threshold range representing the bearing fault feature component was determined based on the results of simulation experiments. However, the feature components of the acquired bearing fault signals should be much more complex than simulated signals. Therefore, we will focus on determining a more appropriate threshold range for acquired signal in future work. In addition, the de-nosing performance of proposed LRPCA method was verified by the signal simulation Equation (20), and Figure 9 shows that it performs better than other methods. In fact, the robustness of the method can be improved by statistical evidence. By extending the evaluation to greater number of rounds, the results of each round are collected, and through meaningful statistical tests, the parameters of the method can be optimized, leading to a significant improvement in noise reduction performance. Therefore, we will also carry out more deep research to that end in future work.

## Figures and Tables

**Table 1:** Parameters in simulated fault feature signal of a bearing’s inner race.

ξ	$\upsilon_{i}$	*I*	*L*	*A*	$B_{i}$	$C_{il}$	$\varphi_{i}$	$\varphi_{il}$	$f_{n}$	$f_{r}$	$f_{c}$
800	0.02/$f_{c}$	250	100	1	0.0004	2/$l^{2}$	$0^{\circ }$	$0^{\circ }$	2000 Hz	30 Hz	125 Hz

**Table 2:** Experiment parameters of fault bearing.

Number of Roller Elements	Roller Diameter (mm)	Medium Diameter (mm)	Contact Angle	Rotation Frequency (Hz)	Fault Frequency (Hz)	Sampling Points	Sampling Frequency (Hz)
z=17	d=8	D=46	$\alpha =14.04^{\circ }$	$f_{r}$ = 7.225	$f_{o}$ = 51.05	*N* = 20,000	$f_{s}$ = 10,000
